# Remission of symptoms is not equal to functional recovery: Psychosocial functioning impairment in major depression

**DOI:** 10.3389/fpsyt.2022.915689

**Published:** 2022-07-26

**Authors:** Hao Yang, Shuzhan Gao, Jiawei Li, Haoran Yu, Jingren Xu, Chenchen Lin, Hua Yang, Changjun Teng, Hui Ma, Ning Zhang

**Affiliations:** ^1^The Affiliated Brain Hospital of Nanjing Medical University, Nanjing, China; ^2^School of Psychology, Nanjing Normal University, Nanjing, China

**Keywords:** depression, psychosocial functioning, whole-course management, function, remission, recovery

## Abstract

The ultimate goal of depression treatment is to achieve functional recovery. Psychosocial functioning is the main component of functional impairment in depressed patients. The concept of psychosocial functioning has an early origin; however, its concept and connotation are still ambiguous, which is the basic and key problem faced by the relevant research and clinical application. In this study, we start from the paradox of symptoms remission and functional recovery, describe the concept, connotation, and characteristics of psychosocial functioning impairment in depressed patients, and re-emphasize its importance in depression treatment to promote research and clinical applications related to psychosocial functioning impairment in depressed patients to achieve functional recovery.

## Introduction

Major depressive disorder (MDD) is a common mental illness imposing a serious burden on individuals and society (1, 2). By 2030, MDD may become one among the diseases with the highest burden on patients in the world (3). The depressive symptoms trigger functional impairment, and functional impairment reduces patients’ quality of life. Patients experience difficulties in important aspects of their personal roles, such as school, work, family, social interaction, and recreation (2), ultimately leading to a heavy personal and social burden of the illness. For decades, symptoms have been considered the primary concern as the most dominant paradox of depression. However, with the improvement of depressive symptoms, a rising number of studies have indicated that patients’ quality of life remains unsatisfactory after remission (4–6) and that asymptomatic patients or those with residual symptoms (7) or subthreshold depression (8) remain significantly function impaired. The elimination of depressive symptoms provides a relatively good improvement in patients’ quality of life but does not completely resolve the problem of recurrence/relapse and long-term low quality of life in depressed patients. Impairment of psychosocial functioning is one of the most important causes, and symptom remission is not equal to functional recovery. Research related to psychosocial functioning has an early origin, but its concept and connotation are still vague, leading to scattered evidence of related studies and making it difficult to summarize its characteristics and patterns validly. It is the key problem hindering its clinical research and application. Depressive symptoms cannot completely reflect the mental health status of depressed patients, and it is difficult to make a sufficiently valid judgment of their quality of life and long-term prognosis. Psychosocial functional impairment is the key challenge for the current efficacy assessment and disease recovery in depression. In this study, we start from the contradiction between the current efficacy assessment and functional recovery, clarify that psychosocial functional impairment is the essence of functional impairment in depression, describe the concept, connotation, and relevant laws of psychosocial functional impairment in depression patients, emphasize its determinant role in the treatment of depression, and promote relevant research and application to recognize the functional recovery.

## Challenges in efficacy assessments

### Efficacy assessments

The components of efficacy assessment of depression treatment are closely related to the understanding of its functional impairment. The early understanding of functional impairment in depression was symptom-related impairment. In 1989, the MacArthur Foundation (9) sponsored a series of conferences aimed at reaching a consensus on the stages of MDD treatment, and in 1991, the 5R criteria were consolidated, and remission was introduced as the critical goal (10) in the acute phase of MDD treatment. Frank et al. [Frank et al. (11)] defined remission as an asymptomatic (number of symptoms of less than or 2) period maintained for more than or 2 weeks but less than 8 weeks. Defining asymptomatic (number of symptoms of less than or 2) period for more than or 8 weeks as recovery, an efficacy assessment strategy based on the number and extent of symptoms has thus been developed. It has standardized the symptom indicators for depression treatment, which had significant implications for depression treatment and became the main evaluation strategy for decades. The main scales used for further specific quantification are Hamilton MDD Inventory (HAMD-17) (12), Beck MDD Inventory (BDI) (13), and Montgomery–Asberg MDD Rating Scale (MADRS) (14). The early definition of recovery has not required functional recovery [ability to work, enjoyment of interpersonal relationships, and overall quality of life (7)]. Subsequently, an increasing number of studies have found that the functional recovery in depressed patients lags behind symptom control (15), and the patients with worse functional recovery have a lower quality of life and higher relapse rates.

Efficacy assessment should also introduce an assessment of patients’ functional status (as shown in Table 1), which, according to the American Psychiatry Association (APA) (16), should include quality of life, ability to work, social integration, social interaction, and satisfaction with interpersonal relationships. The National Institute for Health and Care Excellence (NICE) clinical guideline on depression in adults also emphasizes (17) that the assessment of patients with depression should include symptoms, functional impairment, and interpersonal, and social difficulties associated with them. The NICE 2019 guideline for depression in children and young adults (17) re-emphasizes that efficacy should be inclusive of symptoms, family functioning, and social functioning. The Canadian Network for Mood and Anxiety Treatments (CNMAT) has clearly stated (18) that recovery should be inclusive of symptom remission and functional improvement and that these functions are of limited relevance to depressive symptoms and lag behind symptom improvement, mainly including those aspects of social, occupational, and somatic functioning of the patient. With the increased understanding of the functional connotations of depression, the Royal Australian and New Zealand College of Psychiatrists (RANZCP) (19) clinical practice guidelines state that the goal of depression management has shifted from response through remission to recovery to further building resilience to prevent relapse and recurrence. The functional assessment of symptoms and psychosocial factors before, during, and after the treatment allows for better restoration of function in depressed patients, and early recognition of signs of relapse and recurrence (20) has become a consensus in various guidelines. Additionally, remission is no longer sufficient as the only indicator for evaluating the efficacy of depression in the acute phase. Remission is of epoch-making significance as an assessment index for the acute phase of depression, while the functional assessment should be used as a long-term prognostic index. However, what is the function of depression and how to perform the functional assessment are important issues that are currently faced.

**TABLE 1:** Recommendations for treatment assessment from the guidelines.

Guideline	Year	Maintain treatment	Assessment of efficacy	Advice
CANMAT clinical guidelines	2016	6–9 months/ ≥ 2 years	A total of 80% of treatment studies (247 studies) reported only symptom outcomes	Recovery involves both relief of symptoms and improvement of functioning
WFSBP guidelines	2015	6–9 months/ ≥ 3 years	Symptom assessment	Track mood and early warning signs of relapse or recurrence
RANZCP clinical practice guidelines	2020	6–12 months/ ≥ 1 years	Symptom assessment	Comprehensive assessment and intervention to achieve functional recovery
NICE clinical guideline (Adult)	2009	≥ 6 months	Symptom assessment	Combination of symptom and functional impairment assessment
NICE clinical guidelines (children and young people)	2019	≥ 6 months	Symptom as well as family and social functioning	Stress the assessment of symptoms, functioning, and psychosocial risk in the whole course
APA clinical practice guideline	2019	Balance of benefits *vs.* harms/burdens	Symptom assessment; call for the development of other indicators of efficacy assessment	Consider improvement in a wide range of functional outcomes beyond symptom change

### Challenges of efficacy assessment

The introduction of the 5R definition and criteria has provided an efficacy assessment protocol for treatment aimed at controlling depressive symptoms at that time. It has facilitated the development of strategies for the assessment and management of whole-course management. The introduction of this metric has been groundbreaking, and as the symptoms of depressed patients around the world have gradually improved, achieving functional recovery has become the further goal of antidepressant treatment, and the “symptom era” of efficacy assessment has been challenging.

#### Symptoms are insufficient to evaluate long-term prognosis

Evaluation of disease prognosis should include the patient’s quality of survival and risk of relapse. Using a symptom-based assessment approach to assess the efficacy of antidepressant treatment is immediate and valid, and provides a visual representation of treatment effectiveness regarding the symptom improvement. The assessment allows clinicians to quantify the patient’s disease status related to depressive symptoms and determine the disease progression and prognosis accordingly. However, depression is associated with much more functional impairment beyond depressive symptoms. In 2004, Cieza et al. (21) developed the International Classification of Functioning, Disability and Health (ICF) Core Set for depression to better assess the functional impairment of depressed patients. The Set included 121 core categories covering the following three areas: Physical function, activity and participation, and environmental factors. Symptom-based assessments are incomplete in reflecting the patient’s health status. In a systematic review on functional impairment in depression, McKnight and Kashdan (15) suggested that improvement in social functioning lags behind symptom control [up to 4 years (22)] and that functional impairment is not exclusively symptom related. Therefore, it is inaccurate to assess the patients’ quality of life after remission in terms of “asymptomatic” status.

Symptoms are the main conflict in the acute phase of depression and should be the first concern, but this indicator becomes apparently powerless after the patient has reached remission. Functional impairment is clearly associated with future relapse, and in all three studies reporting social functioning included in the systematic review of risk factors for depression relapse by Prieto–Vila et al. (23), impaired social functioning has been positively associated with depression relapse (24–26). There is also evidence pointing to an association between impairment in family functioning (25), somatic functioning (24), and depression relapse. The functional assessment may reflect both long-term quality of life and risk of relapse. An organic combination of symptom assessment and the functional assessment helps the evaluator to understand the present state and long-term risk of the depressed patient.

#### Inadequate functional assessment

Functioning covers a wide range of content. The current connotation of functional impairment in depressed patients is vague, and there is a lack of correlating assessment norms. The researchers have mainly used alternative methods for assessment, and the tools often used for the alternative assessment are social functioning assessment tool, occupational ability assessment tool, cognitive functioning assessment tool, and quality of life assessment tool. There are often multiple alternative assessment tools for each major category, such as the Short-Form Health Survey (SF-36) (27), the revised Dyadic Adjustment Scale (RDAS) (28), Inventory of Interpersonal Problems (IIP) (29), and Sheehan Disability Scale (SDS) (30) to evaluate social functioning; Work Ability Index (WAI) (31) and Endicott Work Productivity Scale (EWPS) (32) to assess workability; and Mini-Mental State Examination (MMSE) (33), Montreal Cognitive Assessment tool (MoCA) (34), and Clock Drawing Test (CDT) (35) to assess cognitive function. The choice of the assessment tool for a certain functioning is subjective between different studies. For example, in the meta-analysis by Van Leeuwen et al. (36), it was noted that five of the 33 studies included in the analysis assessed patients’ social and occupational functioning using five different instruments. Weissman et al. (37) summarized through a systematic review of the following three most commonly used scales for assessing social functioning: Social Adjustment Scale-Self Report (SAS-SR) (38), Social Adaptation Self-Evaluation (SASS) (39), and SF-36 (27). Which function is selected for assessment and how that function is evaluated are the current dilemma faced on the functional assessment in MDD. The lack of clarity in the connotation and dimensions of psychosocial functioning impairment in depression and the lack of specific assessment tools make the current functional assessment and validity inadequate and insufficient, respectively.

## Why psychosocial functioning?

Functioning, social functioning, and psychosocial functioning are often used in a casual manner, and it is necessary to clarify their connotations. Functioning refers to the beneficial effect played by a thing or method and is often used in disease evaluation to determine the severity of disease damage. Functioning has a very wide range of connotations depending on the subject. In 1974, Strauss and Carpenter (40) argued that in addition to symptoms and duration, social functioning should be added to the diagnostic criteria for schizophrenia as a criterion of severity. Specifically, the patient’s social functioning, work functioning, and hospitalization were to be included in the assessment. Social functioning is an early and widely attended component of mental illness; impairment in somatic, work, and family functioning; and interpersonal relationships in depressed patients has been progressively confirmed as the research on functional impairment in depression has intensified. The requirements for the scope of functional assessment of depression extend beyond social functioning. The *Diagnostic and Statistical Manual of Mental Disorders*, Fifth Edition (DSM-5) (41) requires that depressive symptoms cause clinically significant distress or result in impairment of social, occupational, or other important functions in terms of severity. In the subsequent “Functional Consequences of Major Depressive Disorder,” it is stated that “among individuals in general medical settings, individuals with major depressive disorder have more pain and somatic disorders and exhibit more severe decrements in somatic, social, and role functioning.” Of these, role functioning refers to the individual’s function in personal life in terms of marital, occupational, and social relationships (42). Social and role functions measure the degree of individuals’ functional impairment due to disease damage in terms of social roles, and the subjective feelings of patients should also be emphasized. As understanding of the impact of the disease on patients’ psychological and social functioning and research has advanced, the concept of social functioning has gradually expanded to become psychosocial functioning. Psychosocial functioning is the essence of functional impairment in depressed patients.

## Development and current status of psychosocial functioning in depression

### Development

The quest for the conceptualization and connotation of psychosocial functioning in depressed patients is moving forward toward the patients’ ultimate functional healing. In 1988, Barnett and Gotlib proposed that dysfunction distinguishes depressed patients from healthy individuals (43). The early stages of psychosocial functioning development in depressed patients revolve around the relationship between depressive symptoms and psychosocial functioning. Early researchers conducted a series of studies on functional impairment in depressed patients (44–48), and Barnett and Gotlib (43) classified these functional impairments into etiologic and symptomatic functional impairments based on the relationship between functional impairment and symptoms. They proposed that symptom-induced impairment accounted for only a fraction of psychosocial functioning in depressed patients and hoped to improve psychosocial functioning and achieve disease prevention through early intervention in the etiological functional impairment.

Wells et al. (49) instead focused on the specific effects of impaired psychosocial functioning in depression, emphasizing the importance of patients’ subjective well-being in psychosocial functioning. The researchers specifically explored the relationship between the impaired psychosocial functioning and the depressive symptoms, finding that the depressive symptoms in patients who did not meet the diagnostic criteria could also impair their psychosocial functioning. In 1988, Coryell et al. (50) conducted a 5-year follow-up of psychosocial functioning in depressed patients, noting that the impairment of psychosocial functioning in depression is multifaceted and symptom assessment is a poor substitute for the functional assessment. The persistence of psychosocial functioning in the asymptomatic phase of depression has been subsequently confirmed. Depressive symptoms (physical) and psychological effects on psychosocial functioning are complex, with one part of researchers working to improve symptoms and eliminate their effects on psychosocial functioning and another part focusing on non-symptomatic factors intervening with psychosocial functioning. In 1991, Frank et al. (11) standardized the 5Rs criteria for better improvement of depressive symptoms and clarification of the relationship between psychological and physiological damage and illness. This not only opened a new era of symptom interventions that the research and clinical strategies related to depressive symptom interventions have flourished but also led to the neglect of the non-symptomatic aspects of psychosocial functioning. In the following decade, studies related to psychosocial functioning in depressed patients focused on exploring the relationship between depressive symptoms and psychosocial functioning; for example, In 1998, Miller et al. (51) investigated the efficacy of medication on psychosocial functioning in depression. In 2000, Judd et al. (52) explored the relationship between depressive symptoms and levels of psychosocial functioning. In 2002, Hirschfeld et al. (53) further explored the link between the symptom improvement and the psychosocial functioning improvement and found the unique role of psychotherapy in improving psychosocial functioning in depressed patients. In 2004, Judd et al. (54) found an ameliorative effect of drugs on psychosocial functioning in minor depression. In the process, the pattern of psychosocial functional impairment in depressed patients has become clearer.

Some scholars continue to focus on the non-symptomatic factors of psychosocial functional impairment in depressed patients. Zimmerman et al. (55) has pointed to the importance of “coping skills, general well-being, positive aspects of mental health such as activism, vitality, and self-confidence” as subjectively felt psychological functions in depressed patients. He has conducted a series of studies that called for a re-focus on psychosocial functioning (56–60). In 2007, Malhi et al. (61) linked psychosocial functioning to neuropsychological impairment in patients with bipolar depression and found that executive function, attention, and fine motor memory were closely related to psychosocial functioning in bipolar depression patients. This finding led to the exploration of neuropsychological mechanisms of psychosocial functioning. Hasselbalch et al. (62) suggested that psychosocial functional impairment, quality of life, and risk of relapse could be assessed by evaluating cognitive impairment in depressed patients. A systematic review by Evans et al. (63) confirmed the neuropsychological mechanisms of psychosocial functional impairment. Lam et al. (64) found that subjective cognitive impairment had a greater impact on psychosocial functioning than objective cognitive functioning and called for targeted interventions to promote psychosocial functioning recovery by targeting both subjective and objective cognitive impairment. In the recent years, research has continued to explore the relationship between psychosocial functioning impairment and cognitive functioning impairment in depression, and the development of non-symptomatic dimensions of psychosocial functioning impairment has contributed to the discovery of new treatments.

The focus on psychosocial functioning impairment in depressed patients has its origins in the goal of achieving functional recovery, but it has not received sufficient attention so far. On the one hand, this is due to the focus on depressive symptoms while neglecting psychosocial functioning. On the other hand, it is the long-standing ambiguity of the concept of psychosocial functioning, the lack of uniform criteria for the instruments used to assess psychosocial functioning, and the difficulty of making valid comparisons between studies hindering the exploration of its patterns.

### Current status

The current understanding and application of psychosocial functioning impairment in depression are inadequate. The concept of psychosocial functioning impairment in depression is not sufficiently clear, and functioning is often used interchangeably with psychosocial functioning and psychosocial impairment. This results in scattered research and a lack of valid understanding and summary of its patterns. There are difficulties in the assessment and intervention of psychosocial functioning. The current psychosocial functioning assessment scale for depression has no standards and norms and usually uses the generally applicable social functioning scale, quality of life scale, and occupational functioning scale. Of these, the generally applicable scale can hardly reflect the complete status of functional impairment in depressed patients. Weissman and Bothwell (65) modified the Social Adjustment Scale (SAS) into the Social Adjustment Scale Self-Report (SAS-SR) to evaluate the psychosocial impairment of psychiatric patients to improve the above-discussed situation. The modification emphasized the patient’s subjective experience of role functioning but still did not reflect the subject’s psychological functioning (subjective well-being, “hot” cognition). The most commonly used psychosocial functioning assessment scale for depression today is the SDS (2), which is a generally applicable scale defining SDS of less than or 6 as remission of functioning. With only three items, the SDS has the advantage of being easy to perform, but it is difficult to get a comprehensive picture of the functioning of each domain.

The assessment of psychosocial functioning has also received insufficient attention, and symptom assessment remains the dominant assessment of efficacy, with a meta-analysis by Kamenov et al. (66) noting that 80% of depression studies have had symptom assessments. Patients in subthreshold depression and post-remission have prominent impairment in psychosocial functioning and are at high risk for depressive episodes (67) and relapse (68), but there are no norms for assessing psychosocial functioning in the above-mentioned two disease states, and indicators of long-term outcomes for depressed patients remain undetermined (2). There are also difficulties with interventions for psychosocial functioning. It is currently believed that multiple-receptor antidepressants, such as serotonin and norepinephrine reuptake inhibitors (SNRIs), are better for functional improvement, and duloxetine has optimal efficacy for psychosocial functioning in depression. However, multiple receptors also imply more side effects, and the benefits and risks are difficult to balance (69). Additionally, multiple-receptor antidepressants are not recommended for use in subclinical depression and depression in children and adolescents (16). Moreover, because antidepressants provide limited improvement in functioning after remission, the APA (16) has recommended psychotherapy as the first-line treatment for relapse prevention in depressed patients. There is currently little evidence basis for psychotherapy, and the lack of quantifiable efficacy indicators makes it difficult to conduct evidence-based studies and form valid recommendations.

## Psychosocial functioning and major depression

Impairment of psychosocial functioning occurs throughout the course of depression, arising before the onset of the illness, worsening in the acute phase, gradually decreasing after clinical remission, and exacerbating again after a recurrence or relapse of depression. The reduced and elevated degree of psychosocial functioning in depressed patients is closely related to its influencing factors and has different patterns in the different depression stages. In depression assessment, the symptom assessment defines the disease state, and the psychosocial functioning assessment evaluates the health status. The use of more flexible and comprehensive interventions can help achieve functional recovery (As shown in Table 2).

**TABLE 2:** Difference phases in major depressive disorder.

Phase	Criteria	Aim	Advice
Before MDD (subthreshold depression)	2≤ number of symptoms <5	Disease process delaying	Comprehensive assessment of psychosocial functioning and depressive symptoms, flexible intervention
Acute phase	Number of symptoms ≥ 5, ≥ 2 weeks	Functional impairment reduction	
After remission	Number of symptoms ≤ 2, ≥ 2 weeks	Relapse prevention	

### Before diagnosed

Subclinical depression (subsyndromal, subclinical, subthreshold, or minor depression) has depressive symptoms (≥ 2 or < 5 symptoms) or duration (< 2 weeks) not meeting the diagnostic criteria for depression, and this group is highly heterogeneous with significant functional impairment (70, 71). This group is also at extremely high risk of developing depression (67, 72, 73). A meta-analysis by Lee et al. (74) has noted that people who were subclinically depressed had an approximately 2-fold risk of developing depression compared to those without depressive symptoms. Rodríguez et al. (71) focused on subclinical depression in a systematic review noting that people with subclinical depression have significant functional impairment in daily activities, psychological stress, health perception, work capacity, and somatic functioning. The degree of functional impairment is lower than in depressed patients but still cannot be ignored (71). Considering different populations, subclinical depression in adolescents is prominent in terms of impairment in social–emotional functioning (emotional functioning, shame proneness, and adaptive functioning) (70). Conversely, older adults report more somatic function, cognitive function, and self-perception of health (75–78). Symptom assessment alone in people with subclinical depression hardly reflects the severity of the illness. The combination of psychosocial functioning assessment can better reflect the patient’s mental health status and accordingly provide individualized interventions to delay the disease process (79, 80).

### Acute phase

The acute phase of depression is defined as the stage in which a patient is diagnosed by a semi-structured interview (SCID-I or MINI), based on the DSM-5 (41) or International Classification of Diseases-10 (ICD-10) (81) diagnostic criteria for major depressive disorder (DSM-5) or depressive episode (ICD-10) before reaching remission. Patients in the acute phase of depression have prominent symptoms interacting with psychosocial functioning, and patients have the most severe impairment in quality of life during this phase. Depressive symptoms are closely related to impairment in psychosocial functioning; Vittengl et al. (82), Hirschfeld et al. (53), and Guico–Pabia et al. (83) indicated that depressive symptoms are the key factor in the impairment of psychosocial functioning in the acute phase. Judd et al. (84) followed 371 depressed patients monthly for a total of 10 years and found a parallel relationship between the increase in psychosocial functioning impairment and depressive symptom severity. An improvement of depressive symptoms can rapidly restore psychosocial functioning in the acute phase. However, psychosocial functioning impairments consist of not only depressive symptoms but also cognitive impairment. Weightman et al. (85) systematically analyzed the relationship between cognitive impairment and psychosocial function and concluded that cognitive function is one of the mechanisms of action of psychosocial function, and the related cognitive functioning comprises emotional and empathic performance, subjective cognitive function, and mentalization. However, the composition of psychosocial functioning in acute-phase depression cannot be understood as a simple summation of the impairment of related functions triggered by symptoms and functional cognitive impairment. The interaction between the three factors makes the complex presentation of psychosocial functional impairment. Symptoms play an important role in the impairment of psychosocial functioning in the acute phase of depression, and the combination of psychosocial functioning assessment and intervention remains valuable. The improvement of psychosocial functioning is delayed compared to symptoms (86–88). The combination of pharmacological and other psychosocial interventions can restore function more quickly and improve depressed patients’ quality of life in the acute phase.

### After remission

Reducing relapse rates is a major task for depressed patients after remission. The mainstream clinical guidelines recommend that the depressed patients should maintain antidepressants for at least 6 months after reaching remission to reduce relapse rates (16–20). A meta-analysis by Kato et al. (68) confirmed that relapse was more likely to occur 6 months after discontinuation of the drug. Although studies confirmed that maintenance treatment for 6–9 months can substantially reduce relapse rates, there have been no studies to answer why is maintenance treatment required. We believe that psychosocial functioning may be the most important component of this. Wang et al. (89) found that patients recovered to a more stable level of psychosocial functioning after 6 months of antidepressant maintenance therapy by following depressed patients 12 months after they reached remission. Patients with worse psychosocial functioning at baseline had a higher relapse rate. Psychosocial functioning is closely related to the long-term relapse rate of depression (2), and antidepressants have a limited effect on improving psychosocial functioning at this stage. A meta-analysis by Guidi and Fava (90) for sequential therapy with applied psychotherapy and medication showed that sequential therapy combined with antidepressants or applied alone reduced the risk of relapse and recurrence of depression compared to antidepressants alone. After the depressed patients reach remission, the level of psychosocial functioning can effectively reflect the health status and patients’ long-term prognosis, and comprehensive psychosocial functioning interventions can further improve the long-term prognosis according to their psychosocial functioning status.

## Concepts, dimensions, and traits of psychosocial functioning in depressed patients

### Concepts

Psychosocial functioning is not a concept exclusive to depression; it first appeared in 1963 in the studies on older adults and referred to the ability to perform daily living activities (91). Subsequently, psychosocial functioning entered the field of assessment of various disorders. Psychosocial functioning has different connotations depending on the illness. Over time, as research on psychosocial functioning in depression gradually expanded and deepened, the connotations of psychosocial functioning in depressed patients have gradually emerged. The researchers have proposed their own interpretations based on their knowledge of the patterns of psychosocial functioning in depression. In 1983, Feragne et al. (92) developed the first Psychosocial Functioning Inventory (PFI) for the multifaceted assessment of mental health outcomes in psychiatric patients, focusing on the following components: Patient’s subjective well-being (positive affect, negative affect, and life satisfaction) and social role performance (spouse role, parent role, housemate role, and subjective role performance). This established the basic connotation and scope of psychosocial functioning (i.e., psychosocial functioning) reflects the mental health status of a person, and its scope contains the following two main aspects in psychiatric patients: The subjective feelings in daily life and social functioning. In 2006, Zimmerman et al. (55) explained the psychosocial functioning of individuals with depression, showing that individuals can perform normally in their primary roles, such as being a student, parent, homemaker, or worker.

The above discussion mainly explains psychosocial functioning regarding various aspects of the patient’s functional impairment, which is a brief summary. In 2007, Malhi et al. (61) stated that psychosocial functioning is a complex concept of interactions and activities around personal, occupational, and recreational activities. They also emphasized the interaction between the individual and society. The progressive development and refinement of the concept of impaired psychosocial functioning in depression have been complemented by the discovery of its patterns. In 2011, Lam et al. (93) emphasized that psychosocial functioning in depressed patients must include the following three areas that the patients are most concerned about: Quality of life, and social and occupational functioning. In 2014, Kasen et al. (94) provided a more comprehensive and detailed explanation of psychosocial impairment in depression based on the previous research. According to Kasen et al., the impairment in psychosocial functioning is characterized by impairment in interpersonal relationships, impaired social skills, disturbed personal achievement and occupational roles, negative mood, and psychological stress, which can precede, accompany, or lag the emergence of clinical pathology, especially in depression, and can increase the risk of recurrence and impaired quality of life. The concept of psychosocial function is thus becoming increasingly clear. In 2016, Zhang (95) proposed the first clear definition of psychosocial functioning in depressed patients: the ability of an individual to form functional relationships with others and society in a mutually satisfying manner and to perform their role on their own to accomplish a healthy life. Psychosocial functioning is rooted in function, and a clear definition facilitates the exploration of patterns of functional impairment in depression and the development of assessment and intervention accordingly.

### Dimensions

The discussion of the dimensions of psychosocial functioning impairment in depressed patients should be combined with theoretical derivations and evidence provided by empirical studies. In 1983, Feragne et al. (92) first established the following basic dimensions of psychosocial functioning: Subjective well-being and role functioning. Among these two dimensions, subjective well-being was based on the explanation proposed by Beiser (96) in 1974, stating that the subjective well-being should contain at least the following three factors: Negative affect, positive sense of involvement, and long-term satisfaction. Role functioning evaluates the individual’s functioning in common social roles, such as spouse, parent, and housemate. Zimmerman et al. (55), Malhi et al. (61), and Lam et al. (97) explained the psychosocial functioning of depressed patients, all falling under the category of role functioning. Kasen et al. (94) also incorporated negative mood. In 2012, Cabello et al. (98) conducted a systematic review of the characteristics of psychosocial functioning impairment in depressed patients, classifying the functional impairments reported in the included studies according to ICF. Emotional function problems, energy and drive functioning problems, pain, cognitive problems, relationships with people, and work problems were the most frequently reported. Among them, emotion function problems belong to the category of subjective well-being, interpersonal and work problems belong to role functioning, energy and motivation functions and pain are included in somatic functioning, and cognitive problems belong to cognitive functioning.

The above-mentioned functional impairments were reported based on their respective measurement instruments. The instruments used for the functional assessment in depression research mainly use the general applicability scales, except for SASS, which is specific to depression. According to the content, they can be divided into the following: General functional assessment scales, quality of life scales, occupational functioning scales, and well-being scales. In 2019, Krause et al. (99) conducted a systematic review of studies related to depression outcomes in adolescents (12–19-year old) from 2007 to 2017, in which overall functioning (48 of 92), cognitive processing (9 of 92), interpersonal relationships (6 of 92), personal growth (dealing with mood, thoughts, feelings; assertiveness; attitude toward self; and autonomy) (7 of 92), service use and satisfaction (8 of 92), and quality of life (7 of 92) were assessed more frequently, and only a few studies assessed physical health (3 of 92). Among the above-described functional impairments, overall functioning and interpersonal relationships belong to the category of role functioning, cognitive processing belongs to cognitive functioning, personal growth can be classified as personal well-being, and service use and satisfaction can reflect patients’ functional status from their dependence on assistance and services (100, 101).

Quality of life reflects the individual’s subjective satisfaction with their functioning (102). In 2017, Sheehan et al. (2) conducted a systematic review of functional assessment scales used in research related to adult depression (18–65-year old). These instruments can be divided into the following four categories: General functioning [Global Assessment of Functioning (GAF) (103), Longitudinal Interval Follow-up Evaluation-Range of Impaired Functioning Tool (LIFE-RIFT) (104), Multidimensional Scale of Independent Functioning (MSIF) (105), SDS, SAS-SR (39), WHO Disability Assessment Schedule (WHODAS) (106), Work and Social Adjustment Scale (WSAS) (107)]; quality of life [Euro QoL 5-Dimension Questionnaire Visual Analog Scale (EQ-5D VAS) (108), Quality of Life Enjoyment and Satisfaction Questionnaire (Q-LES-Q) (109), Short- Form Health Survey (SF) (27)]; occupational functioning [Work Productivity and Activity Impairment questionnaire (WPAI) (110), WHO Health and Work Performance Questionnaire (HPQ) (111)]; and well-being (WHO Well-Being Index (WHO-5) (112)]. In 2018, Bingham et al. (113) conducted a systematic review of the functional assessment in older adults with depression (≥ 60 years of age). They categorize the functional measurement tools based on what they measure. These tools are classified as the following, Instrumental Activities of Daily Living (IADL): The Performance Assessment of Self-Care Skills (PASS) (114) and the Lawton & Brody IADL scale (Lawton & Brody) (115); social functioning instruments such as SAS and the Duke Social Support Index (DSSI) (116); and both contained instruments such as the Late-Life Function and Disability Instrument (LLFDI) (117) and WHODAS (118). Instrumental Activities of Daily Living refers to the individual’s ability to perform adaptive activities in their environment, such as shopping, cooking, housework, cleaning, using transportation, making phone calls, and managing medication and money (119), and is used to determine the individual’s basic social adaptation skills (120). Instrumental Activities of Daily Living and self-care reflect the realization of the individual’s basic roles and should be classified as role functions. Subjective well-being was classified into “affect balance” and “life satisfaction” according to Morton Beiser’s interpretation. The general interest belongs to positive emotions; thus, it should be classified as affect balance.

The above-mentioned scales have been dimensionally analyzed, as shown in Table 3. In the three populations of adolescents, adults, and older adults, the main dimensions of functional assessment have been focused on role functioning, and subjective well-being has been partially addressed on some scales (SAS-SR, LIFE-RIFT, GAF, Q-LES-Q, SF, and WHO-5). Some scales have assessed symptoms (GAF, EQ-5D VAS, and SF), and WHODAS, EQ-5D VAS, Q-LES-Q, SF, HPQ, and WHO-5 have assessed somatic functioning. The above-mentioned scales are deficient in dimensionality when applied to the assessment of psychosocial functioning in depression. On the one hand, the content of subjective well-being is missing/absent. On the other hand, the generally applicable scales hardly recognize the cognitive impairment in depression. In the recent years, researchers have classified depressive cognitive functions as “cold cognition” and “hot cognition.” Cold cognition is mainly related to neurocognitive functions, such as arithmetic, comprehension, executive function, attention, and memory. Hot cognition is the individual’s attitude toward oneself, the environment, and the future, such as negative automatic thoughts and negative perceptions (121). Hot cognition reflects the psychological functioning of the patient and should be included in the psychosocial functioning of depression (122). In contrast, the neurocognitive impairment represented by cold cognition is considered one of the mechanisms of psychosocial impairment in depressed patients, similar to depressive symptoms, which is an influencing factor of psychosocial functioning rather than a performance of psychosocial functioning. Therefore, it should not be considered a dimension of psychosocial functioning in depressed patients. Additionally, somatic functioning was originally a dimension established for patients with organic disorders when the questionnaire was designed for general application, and these impairments have been considered to be related to depressive symptoms and should also be excluded, although the impairments in somatic functioning have been reported in some studies. In 2022, Zhang et al. (122) developed a psychosocial functioning questionnaire for depressed patients, measuring three dimensions, including psychological cognition, subjective well-being, and social functioning. Its dimensional coverage is comprehensive and targeted, which can accurately and adequately reflect the psychosocial functioning status of depressed patients.

**TABLE 3:** Dimensional settings of scales commonly used for proxy assessment.

Category	Scale	Role functioning	Affect balance	Satisfaction	Cognitive functioning	Symptoms	Physical functioning
General functioning	WSAS	Work, household, leisure activities, relationships					
	WHODAS	Self-care, interacting with others, life activities/responsibilities, participation			Understanding and communicating		Mobility
	SDS	Work/studies, social life, family life/home responsibilities					
	SAS-SR	Daily tasks, leisure, hobbies, interpersonal relationships (family, external), social compliance, social behavior, self-perception		Performance satisfaction			
	SAS	Work, social and leisure activities; relationship with extended family; marital role; parent role; and role as a member of a family unit					
	PASS	IADL					
	MSIF	Work, education, residential					
	LLFDI	Social role, personal role, management role, IADL					
	LIFE-RIFT	Work, relationships, leisure		Overall satisfaction with functioning			
	Lawton & Brody	IADL					
	GAF	Relationships, work		Overall satisfaction		Symptom	
	DSSI	Social network, social interaction, instrumental social support, subjective social support					
Quality of life	EQ-5D VAS	Self-care, usual activities				Anxiety/ depression	Mobility, pain/discomfort
	Q-LES-Q	Leisure, relationships, general activities		Overall enjoyment and satisfaction			Physical health
	SF	Physical role functioning, social functioning	Emotional role functioning			Mental health	Physical functioning, bodily pain, general health, vitality
Occupational functioning	WPAI	Work					
	HPQ	Work					Physical health
Well-being	WHO-5		Positive mood, general interest				Vitality

### Traits

#### Persistence and prevalence

The impairment of psychosocial functioning in depressed patients has a persistent nature, as it may appear before the onset of depression, accompany depressive symptoms, and persist long after the attainment of remission. Since 1991, when remission was introduced as a treatment target in the acute phase of depression, clinical experts and patients have found that functional impairment persists after remission. Koen Demyttenaere et al. concluded that patients with HAM-D17 of less than or 7 were adequate regarding remission of negative emotions, but the patients showed poor improvement in positive emotions, hedonic, functional, or meaningful life (123). Sheehan et al. (2) conducted a systematic review targeting recovery of depressive functioning, highlighting once again the persistence of psychosocial functional impairment in depressed patients after remission. Furthermore, as previously mentioned, both subthreshold depression and the acute phase of depression are accompanied by significant functional impairment, which jointly characterize the persistence of psychosocial functioning impairment in depression. Psychosocial functioning impairment in depression has characteristics of pervasiveness. Kessler et al. (124) followed 335 depressed patients over 12 months, 96.9% of whom had psychosocial functioning impairment (SDS). Judd et al. (125) reported that 87.1% of 964 person-months at the MDD level were rated as poor or very poor in overall functioning [Longitudinal Interval Follow-up Evaluation (LIFE)].

The persistence and prevalence of psychosocial functioning impairment in depressed patients are dependent on their influencing factors (depressive symptoms, cognitive function, and stress). First, depressive symptoms are closely related to psychosocial functioning. Depressive symptoms in all disease stages can impair occupational functioning (64), and symptoms are considered the most influential factor in psychosocial functioning in the acute phase of depression (53). Depressive symptoms can occur in all depression stages and cause psychosocial functioning impairment throughout the depression course. Specifically, subthreshold depressed patients show a “step-wise” relationship between depressive symptoms and psychosocial functioning (84), with a parallel relationship between acute-phase symptoms and improvement in psychosocial functioning (125). Residual symptoms can also significantly impair psychosocial functioning (84). Second, cognitive functioning impairment is closely related to psychosocial functioning impairment in depression (63, 97, 126). Executive function, attention, and memory are the most well-documented cognitive functions associated with psychosocial functioning and are thought to be one of the mechanisms underlying psychosocial functioning impairment in depression (127). Cognitive functioning impairment is prevalent in depression (128, 129) and has persistent features (127). Additionally, systematic reviews have noted that cognitive functioning impairment occurs in the subthreshold depression (72) and the acute phase of depression and persists into the post-remission period (130), with persistent interference with psychosocial functioning. Third, persistent impairment in psychosocial functioning causes psychosocial stress, and the two interact with each other. Impaired psychosocial functioning reduces the ability of depressed patients to cope with stressful events. Patients lack a subjective sense of well-being, feel more negatively regarding stress, and face more severe psychological stress. Patients have difficulty maintaining intimate relationships and general interpersonal interactions, lacking support systems in the face of stressful events (131–133). The patient’s ability to learn and work becomes impaired, leading to a poor performance in school and work, which causes a poor economic–social status. Impaired psychosocial functioning exposes depressed patients to more stressful events and poorer ability to cope with them.

#### Increasing the risk of relapse/recurrence

Psychosocial functioning impairment is an important factor in the relapse and recurrence of depression. A study by Waguih William Ishak et al. found a 2.5–4.1-fold increase in relapse rates in clinically remitted [the Quick Inventory of Depressive Symptomatology-Self Report (QIDS-SR) score of less than or 5] depressed patients with impaired self-rated functioning and/or examiner-rated functioning (134). Prieto–Vila et al. (23) and Hardeveld et al. (135) conducted systematic reviews of risk factors for depression recurrence, in which social functioning, family functioning, occupational functioning, the perception in general health, and recreational functioning were all strongly associated with increased rates of depression recurrence/relapse (25, 26, 136, 137). Poor psychosocial functioning and early not remission of psychosocial functioning are both risk factors for the recurrence of depression. Backs–Dermott et al. (138) reported how the relapsed group in their study had lower levels of psychosocial functioning at baseline and 12 months than the non-relapsed group. Findings from Wang et al. (89) also supported this finding, and further analysis indicated that the patients with poor recovery of psychosocial functioning in the first 2 months after reaching remission were at higher risk of relapse. Jha et al. (139) found that patients with an early improvement in psychosocial functioning in the first 6 weeks of antidepressant treatment were 3–6 and 2–3 times more likely to achieve remission after 3 and 7 months, respectively, than controls. Impairments in social and family functioning weaken the support systems, impairments in health perceptions and occupational functioning reduce economic-social status, and impairments in recreational functioning reduce the subjective well-being. These factors reduce the patient’s ability to cope with stressful events (140), leaving the patient in constant chronic psychosocial stress, eventually resulting in relapse and recurrence.

#### Impairing the quality of life

The WHO (102) emphasizes that the elements of quality of life need to include the following: (1) Subjective experiences, (2) cultural, social, and environmental contexts, and (3) assessment of specific components of life, such as health status, work, family, social connections, and leisure activities. Impaired quality of life is a common and prominent feature of depressed patients. Vaillant et al. (141) conducted a 50-year follow-up study of 19 patients with depression. The patients continued to be disturbed by tobacco and alcohol abuse after remission and had social and marital adjustment difficulties until the age of 68 years, with only three patients having stable marriages. Psychosocial impairment in patients with depression limits the patient’s ability to perform important aspects of daily functioning and makes them perceive their health negatively, resulting in a subjectively experienced decrease in the quality of life (142). In 2012, Cabello et al. (98) conducted a systematic review of psychosocial difficulties faced by depressed patients, artificially classifying the quality of life impairments into somatic functioning (emotional functioning, energy and motivation, cognitive impairment, temperament and personality functioning, sleep disturbance, psychopathological symptoms, and pain) and activity and participation (interpersonal relationships, work problems, self-care problems, and social life problems), with emotional functioning, energy and motivation functioning, cognitive problems, occupational, interpersonal, and social life problems being considered the most common psychosocial difficulties in depressed patients. Impairment of psychosocial functioning causes a decrease in the quality of life at all the stages of depression. Except for the acute phase of depression and post-remission, patients with subclinical depression have a lower quality of life than healthy individuals but better quality of life than those with depression (8, 143). Decreased quality of life can lead to impaired social processes in children and adolescents, setting the stage for subsequent stunting in their development (144). It makes the prognosis worse in older people, complicating recovery obtainment and presenting more co-morbidities and death (145).

Impairment in psychosocial functioning is strongly associated with decreased quality of life, but impairment in psychosocial functioning is not equivalent to an impairment in the quality of life. Teng et al. (146) conducted a meta-analysis of the effect of antidepressants on function and quality of life in children and adolescents and noted that medication improved function but not the quality of life. Hofmann et al. (147) focused on the efficacy of quality of life in depressed patients, finding that medications alone did not improve the quality of life, whereas improvement in quality of life occurred in depressed patients receiving either the cognitive behavior therapy (CBT) alone or CBT combined with medications. A preliminary explanation for this has been provided by Lopes et al.’s (148) moderating mediator model, namely, “information processing speed” moderates the partial mediating effect of “productivity despite sickness presence” on depressive symptoms and quality of life, meaning that neurocognitive impairment may moderate the mediating role of occupational functioning on depressive symptoms and the quality of life. Additionally, the quality-of-life evaluations stress the individual’s subjective attitudes toward dysfunction. Negative cognition, which is one of the characteristics of automatic thinking in depressed patients, can persist beyond the depressive episode (149) and persistently affect the patient’s evaluation of functional impairment, leading to a reduced quality of life.

## Assessment of psychosocial functioning in depression

There has been a lack of consensus on the assessment of psychosocial functioning in depressed patients due to the ambiguity of the concept and connotation of psychosocial functioning. In 2017, Sheehan et al. (2) performed a systematic review of assessment tools applied in previous literature studying functional impairment in depression, and the SDS, Q-LES-Q, and SAS-SR were the most frequently used scales. Bingham et al. (113) reported that the most common scales used to assess psychosocial functioning in older adults were the 36-Item Short Form Survey (SF-36), WHODAS (118), and SDS. Children and adolescents are assessed by the Children’s Global Assessment Scale (CGAS) (150), GAF (103), and Pediatric Quality of Life Enjoyment and Satisfaction Questionnaire (PQ-LES-Q) (109). The above-mentioned scales can be divided by their scope of application into general applicability scales, general applicability scales for psychiatric disorders, and depression applicability scales. The general applicability scales are applicable to all populations and cover the basic components of functioning/quality of life, but in the face of specific disorders, these scales are difficult to adequately reflect all functional impairments of the disorder.

The SDS, GAS (GAF), and CGAF are general functioning assessment scales that are easy to assess and apply clinically, as shown in Table 4. Among them, SDS and CGAF cover the following basic aspects of social functioning: Work/study ability, family functioning, and interpersonal relationships (151). The GAF (GAS) also includes “positive mental health” and symptoms. All three scales are self-assessment scales, which can better reflect the subjective experience of patients. However, SDS and CGAF lack the evaluation of the subjective well-being of individuals (92), and GAS (GAF) lacks the evaluation of the characteristic hot cognitive function (irrational ideas and negative thinking) impairment (152) in depressed patients. The SAS-SR and WHODAS are more detailed and comprehensive in assessing social functioning. Particularly, WHODAS includes self-rating and examiner-rating in the assessment method, and combined with the interview, it can reflect both subjective and objective functioning of patients and the gap between subjective and objective feelings. However, both of them also lack the content related to subjective well-being and hot cognition. Additionally, the questions related to self-care and mobility in the WHODAS are mainly found in severe depression with psychomotor retardation (153) and are not applicable to the functional assessment of general conditions. Both Q-LES-Q and the SF-36 are the quality-of-life assessment scales reflecting individual satisfaction with life and social functioning. However, these scales also lack the hot cognitive assessment. The additional somatic-related questions and the excessive number of questions may cause stress to the participants during the test. Some studies have suggested that the basis of psychosocial impairment is neurocognitive function and advocated for the use of neurocognitive functioning assessment as a substitute for psychosocial functioning status (85, 127). However, neurocognitive functioning assessment items are complex, have certain requirements for the subject’s state at the time, are difficult to perform routinely, and ignore the patient’s self-perception. In 2022, Zhang et al. (122) developed the Psychosocial Functioning Questionnaire (PFQ), which is the first psychosocial functioning questionnaire for depressed patients. This 18-item instrument is easy to apply and contains “subjective well-being,” “psychological cognitive functioning (self-evaluation, self-control, beliefs, and expectations),” and “social functioning,” with comprehensive coverage and good reliability and validity. It is an ideal tool for assessing the psychosocial functioning of depressed patients.

**TABLE 4:** The main proxy tools for assessing psychosocial functioning in adolescents, adults, and older adults with depression.

Category	Instrument	Year	Scope of application	Type	Domains
General functioning	SDS	1983	Generally applicable	Self-rating scale	3 items: work/studies, social life, and family life/home responsibilities
	SAS-SR	1976	Generally applicable	Self-rating scale	48 items within 6 domains: work (paid, homemaker, student); social and leisure activities; relationship with extended family; marital role; parent role; and role as a member of a family unit.
	WHODAS	2004	Generally applicable	Examiner-rating scale	36 items within 6 domains: cognition, mobility, self-care, interpersonal interactions, life activities [domestic responsibilities, leisure, work], and participation in society)
	GAF (GAS)	1976	Generally applicable	Examiner-rating scale	1 item (1–100 point): social/interpersonal, occupational, psychological (e.g., satisfaction), and psychiatric functioning (e.g., symptoms)
	CGAS (Evolved from GAS)	1983	Generally applicable in children	Interviewing + examiner-rating + self-rating	1 item (1–100 points): functioning at home, at school, and with peers
	PFQ	2022	Depression applicable	Self-rating scale	18 items in 3 dimensions: subjective well-being, psychological cognitive functioning (self-evaluation, self-control, beliefs, and expectations), social functioning
Quality of life	Q-LES-Q (PQ-LES-Q)	1993 (2006)	Mental disorders	Self-rating scale	93 (15) items in 6 dimensions: somatic health, subjective feelings, leisure activities, social relationships, general activities, and satisfaction with medication and life
	SF-36	1992	Generally applicable	Self-rating scale	36 items in 8 dimensions: physical function, physical role function, somatic pain, general health, vitality, social function, emotional function, mental health

## Interventions for psychosocial functioning in depression

The reported interventions related to psychosocial functioning in depression are classified as pharmacotherapy, psychotherapy, and physiotherapy. Pharmacotherapy and physiotherapy exert their efficacy mainly through biological influences. Therefore, the interventions are classified here as biological and psychological treatments.

### Biological therapy

#### Medications

Pharmacotherapy is effective in improving psychosocial functioning in depressed patients and is currently considered to act in the following two ways: Reducing depressive symptoms and improving neurocognitive function. As previously stated, any degree of depressive symptoms can lead to a decrease in psychosocial functioning, and the improvement of psychosocial functioning by antidepressants parallels the severity of depressive symptoms (2). Antidepressants with norepinephrine receptor effects have been shown to better improve executive functions (154) related to cognition, vitality, and intelligence while having better efficacy on psychosocial functioning. In 2021, Cao et al. (69) conducted a network meta-analysis (NMA) of pharmacological treatment to improve psychosocial functioning (SDS), with the efficacy ranked in the following order: Paroxetine, levomilnacipran, venlafaxine, quetiapine, desvenlafaxine, agomelatine, escitalopram, amitriptyline, bupropion, sertraline, vortioxetine, and fluoxetine. Depressive symptoms are important and prominent component of psychosocial impairment, and neurocognitive impairment is also considered a pathological mechanism of psychosocial impairment. Therefore, pharmacological interventions are necessary. However, pharmacological treatment is also inadequate. Psychological functions, such as the patient’s subjective perception of emotional balance and well-being, are difficult for biological interventions to influence. Studies have confirmed that the depressed patients still need maintenance treatment for more than 6 months after reaching remission to reduce the relapse rate (68). Zhou et al. (155) noted that the patients with depression had an increased risk of relapse after discontinuation of medication, regardless of the length of maintenance treatment.

How to cope with life stress in a renewed manner and achieve a balance within and outside the individual is a necessary step in the recovery of the patient’s psychological function. Biological and psychosocial factors interact with each other, similar to the pathophysiological process of fracture healing. After the formation of new bone at the fractured part, it is necessary to undertake pressure with limb activities and weight-bearing; thus, the bone scab on the stress axis is continuously strengthened, and the reconstruction of the bone trabecular structure is completed to realize the recovery of fracture. Antidepressants provide mainly a supportive role in the process of functional psychosocial recovery, representing a passive, unconscious intervention in which patients have difficulty acquiring skills to cope with psychosocial stress and achieve functional recovery.

#### Physiotherapy

Physiotherapy has been less studied in relation to improving psychosocial functioning in depressed patients. Electroconvulsive therapy (ECT) improves processing speed, working memory, and part of executive function related to psychosocial functioning after 15 days (156). Repetitive transcranial magnetic stimulation (rTMS) is ineffective for executive functioning but may improve psychosocial functioning by reducing depressive symptoms (157).

### Psychotherapy

Psychotherapy is recommended as the first-choice treatment (16) for mild depression, subthreshold depression, and depression in children and adolescents. It is of great importance to improve the psychosocial functioning of depressed patients. Psychotherapy has currently a poor evidence basis; for example, studies by Zhou et al. (158), Renner et al. (159), and Ward et al. (160) all concluded that cognitive CBT provides clear improvements in psychosocial functioning, while meta-analyses by Monferrer et al. (161), Hunot et al. (162), and So et al. (163) failed to draw conclusions due to small sample size and insufficient quality. It is currently believed that cognitive training (164, 165) and cognitive remediation (166) influence psychosocial functioning by improving cognitive functioning. Interpersonal therapy (IPT) is prominent in adolescent depression (167, 168) and has a long-lasting effect, with some studies showing that such improvements last at least 1 year (168). However, older individuals find it difficult to show the advantages of psychotherapy alone (99) because of complex age-related problems (physical illness, loneliness, and grief), and collaborative care is more effective in restoring psychosocial functioning in such individuals (169). Multiple studies have confirmed that psychotherapy has a longer-lasting effect than antidepressants in preventing the recurrence and relapse of depression (170–172). We believe that psychosocial functioning plays an important role in this. Psychotherapy enhances the patient’s ability to cope with psychosocial stress, as CBT emphasizes teaching the patient skills, such as identifying automatic thoughts and behavioral activation, that can continue to function and have an impact on the patient’s functioning after psychotherapy ends (173). The patients treated with IPT gain the ability to cope with interpersonal relationships and sustain improvements in social functioning (168). Psychotherapy is an important complement to medications, with medication maintenance providing support and psychotherapy enabling faster and better recovery of psychosocial functioning.

### Combination of pharmacotherapy and psychotherapy

The efficacy of combined interventions, including both pharmacotherapy and psychotherapy, is controversial. Kamenov et al. (174) conducted a meta-analysis of relevant studies regardless of age group and shown that the combined treatment had a small but significant advantage over the application of psychotherapy or pharmacotherapy alone regarding the improvement of quality of life and functioning. Cox et al. (175) conducted a meta-analysis on the child and adolescent population, but it was inconclusive due to the paucity of evidence, with only one study reporting a mild advantage of combination therapy over pharmacotherapy alone at 12-month follow-up (176). In contrast, a systematic review of persistent depressive disorder in adults was similarly unable to draw valid conclusions due to the paucity of relevant studies (177). Nieuwenhuijsen et al. (178) showed that drug-combination occupational therapy may mildly improve occupational function, but the long-term efficacy was not significant.

## Summary and prospect

Psychosocial functioning impairments in depressed patients are pervasive and persistent, preventing the patients from functioning effectively in various aspects of daily life, affecting the way they view themselves and their circumstances, and causing negative emotional experiences and decreased subjective well-being. These experiences contribute to the patients’ ongoing impaired quality of life and cause an increased risk of depression recurrence and relapse. Symptom remission is not equal to functional recovery, and symptom assessment alone does not provide a complete picture of the patient’s actual psychosocial functioning status, the paradox of which is even more apparent in depressed patients after remission. Psychosocial functioning is not synchronized with the recovery of depressive symptoms, and functional recovery is delayed compared to symptoms. We suggest that psychosocial functioning assessment should be incorporated into the assessment of long-term outcomes and that management strategies and methods aimed at functional recovery should be set according to the patient’s psychosocial functioning status. The evidenced-based basis for psychotherapy is poor, and the inadequate development of efficacy measurement tools for psychotherapy is a key contributing factor. The current use of tools for assessing the efficacy of psychotherapy is confusing, with some psychological functioning-targeting psychotherapies using social functioning questionnaires to assess the function of a patient or being generalized to use overall functioning questionnaires. Although psychological functioning, social functioning, depressive symptoms, and quality of life influence each other, this effect may be delayed (159, 179), making it difficult for existing assessments to accurately reflect the related efficacy (180). We call for the application of more comprehensive and targeted psychosocial functioning assessment tools to evidence-based research and individualized interventions in psychotherapy to promote the application of psychotherapy in improving psychosocial functioning in depressed patients. In this article, the mainframe and components of the psychosocial functioning of depressed patients are sorted to attract attention and promote relevant research and applications. However, it is not comprehensive and in-depth enough in addressing specific issues. We will concentrate on specific doubts for systematic review and meta-analysis going forward. In the future, the mechanisms underlying the impairment of psychosocial functioning in depressed patients should be further explored, and more relevant interventions should be developed to meet the treatment needs of different conditions. The goal of depression treatment should be more than the absence of symptoms, i.e., achieving functional recovery and restoring the mental health of the individual.

## Author contributions

NZ: original idea. HM: supervision. All authors contributed to the article and approved the submitted version.
